# Development and Validation of LAMP Assays for Distinguishing MPXV Clades with Fluorescent and Colorimetric Readouts

**DOI:** 10.3390/bios15010023

**Published:** 2025-01-06

**Authors:** Nazente Atceken, Sara Asghari Dilmani, Ahmed Choukri Abdullah, Mutlu Sarıkaya, Defne Yigci, Gozde Korkmaz, Savas Tasoglu

**Affiliations:** 1 School of Biomedical Sciences and Engineering, Koç University, 34450 Istanbul, Turkey; natceken@ku.edu.tr (N.A.); sdilmani23@ku.edu.tr (S.A.D.); 2 Koç University Translational Medicine Research Center (KUTTAM), Koç University, 34450 Istanbul, Turkey; gkorkmaz@ku.edu.tr; 3 School of Medicine, Koç University, 34450 Istanbul, Turkey; dyigci21@ku.edu.tr; 4 Department of Mechanical Engineering, Koç University, 34450 Istanbul, Turkey; aabdullah22@ku.edu.tr; 5 Department of Biochemistry Faculty of Pharmacy, Ankara University, 06560 Ankara, Turkey; mutlu.sarikaya@protonmail.com; 6 Koç University & Is Bank Artificial Intelligence Center (KUIS AI), Koç University, 34450 Istanbul, Turkey; 7 Koç University Arçelik Research Center for Creative Industries (KUAR), Koç University, 34450 Istanbul, Turkey; 8 Boğaziçi Institute of Biomedical Engineering, Boğaziçi University, 34684 Istanbul, Turkey

**Keywords:** loop-mediated isothermal amplification technology, monkeypox virus clade detection, novel reverse-duplex detection of amplification by release of quenching probe

## Abstract

Human monkeypox (Mpox) is a zoonotic disease caused by the Monkeypox virus (MPXV). As of 14 August 2024, the World Health Organization (WHO) has declared it a global health emergency. For Mpox, this was the second public health emergency of global significance in the past two years. MPXV belongs to the *Poxviridae* family and is phylogenetically and epidemically divided into two clades: the Congo Basin (Clade-I) and the West African (Clade-II) clades. Clade-I has been associated with more severe disease progression and higher mortality compared to Clade-II, and thus the differentiation between clades can play an important role in predicting disease prognosis. The LAMP technique has the advantages of not requiring thermal cycling and achieving higher amplification in a shorter time compared to qPCR. Different types of LAMP assays were developed in this study to benefit from these advantages. We report the development of LAMP-1 and LAMP-2 assays using the LAMP method to detect MPXV Clade-I and Clade-II, respectively. The LAMP-1 assay includes both fluorescence and visible colorimetric readout tests developed with sensitivities of 10^3^ and 10^7^ copies, respectively. For the LAMP-2 assay, a probe-based test utilizing the Novel R-Duplex DARQ probe was developed, offering fluorescence detection at a sensitivity of 10^3^ copies. As a result, we successfully developed three highly specific molecular diagnostic tests that distinctly differentiate between MPXV clades, delivering essential tools for the precise diagnosis and effective control of Mpox.

## 1. Introduction

Human monkeypox (Mpox) is a zoonotic disease caused by Monkeypox virus (MPXV), a member of the *Orthopoxvirus* genus within the *Poxviridae* virus family [1]. This family also includes the Variola virus, which causes smallpox; the Cowpox virus, which causes cowpox; and the Vaccinia virus, which is used in smallpox vaccine production [2]. MPXV has genetic and epidemiological similarities with members of the same family [3]. This may cause symptomatic similarity and difficulties in diagnosis. The MPXV was first detected in 1958 during an outbreak of smallpox-like illness in monkey colonies held in captivity for research, leading to the naming of this disease ‘monkeypox’ [4]. Despite its name, the natural reservoirs of the disease are not limited to monkeys and include rats, dormice, and other non-human primates [5]. However, the WHO has renamed the virus Mpox as of 2023, out of concern that associating outbreaks and newly identified agents with geographical regions or animal names may cause labeling [6]. The first human case of Mpox disease was reported in 1970 [7]. The virus can infect humans via transmission from animal to human (zoonotic) or from human to human [8]. MPXV can be transmitted between humans through respiratory droplets, contact with other body fluids, or direct contact with lesion material from infected individuals [9]. Zoonotic transmission can occur through direct inoculation through bites and scratches, as well as through contact with the body fluids of infected animals during activities such as hunting, carcass preparation, or playing with animals [10,11,12]. Mpox-infected individuals typically present symptoms similar to those induced by smallpox. After an asymptomatic incubation period of about one to two weeks, infected individuals develop a fever and fatigue, soon followed by a widespread rash [13]. While symptoms such as painful lymphadenopathy (which is rarely seen in other poxvirus infections) can help clinically differentiate Mpox from other poxvirus infections, most other prodromal symptoms are non-specific [14].

For decades, Mpox was an endemic disease with only sporadic cases occurring on the African continent [8]. However, an increase in Mpox cases has been recorded globally in patients who have had no direct contact with endemic regions, attracting global attention [8]. On 14 August 2024, the WHO declared a public health emergency of international concern, fueling efforts to develop strategies to contain the spread of this disease [15]. A state of emergency was previously declared in July 2022, and this is the second such situation in two years. An important component of achieving control over the spread of disease relies on the development of accurate diagnostic tools that can be employed rapidly and without requiring complex infrastructure. A recent outbreak of Mpox in the Democratic Republic of the Congo resulted in the highest number of suspected cases ever recorded, exceeding 20,000 cases and leading to more than 1000 deaths since January 2023 [3]. As there is no FDA-approved treatment method for Mpox, the best strategy is to prevent the spread of infection, emphasizing the need for the early diagnosis of MPXV [3].

MPXV is a double-stranded DNA virus [16]. Genome sequencing and clinical–epidemiological analyses of MPXV isolates have revealed that this virus is divided into two distinct clades [2]: the Congo Basin (Clade-I) and West African (Clade-II) clades. The Congo Basin clade has historically been associated with more severe disease and is thought to be more contagious [2]. There is a geographical division between the two clades, with the Congo Basin clade typically being found in Central Africa and the West African clade usually occurring in West Africa [2]. It is also divided into Clade-I (Clade-Ia and Clade-Ib) or Clade-II (Clade-IIa and Clade-IIb) subclasses [17]. The 2022 outbreak of Mpox corresponds to Clade-IIb, while the 2024 outbreak represents Clade-Ib subclasses [18]. However, in the event of a global spread, the geographical boundaries of these two clades may shift. Therefore, accurately distinguishing between the two clades is crucial for monitoring the spread and impact of this disease.

For many years, point-of-care (PoC) tests have been essential [19]. These tests include paper-based assays, microfluidic systems, portable devices, and technologies that enable results to be monitored via mobile phones [20,21,22]. PoC tests offer several advantages, including reliability, ease of access, minimal need for expert personnel, and inexpensiveness [23]. Isothermal amplification technologies have emerged as valuable tools for diagnosing infectious diseases and detecting pathogens, particularly because they are well suited for PoC applications [23,24,25]. The loop-mediated isothermal amplification (LAMP) method can amplify nucleic acids at a constant temperature, eliminating the need for the thermal cycling steps required in the gold-standard method, polymerase chain reaction (PCR) [26]. LAMP technology is highly sensitive, straightforward, and rapid, making it ideal for nucleic acid detection, even in areas with limited infrastructure [27]. This method involves forming amplicons with a multi-loop structure using a total of six primers—two inner, two outer, and two loop primers—that target eight regions within the gene sequence of interest [27]. LAMP technology serves as a versatile, next-generation detection method for infectious pathogens, mutation analysis, methylation studies, and miRNA detection [23,25,27]. A LAMP amplification product can be detected fluorescently and visually through colorimetry [24,28]. However, due to the use of six primers targeting eight nucleic acid synthesis sites, there is a risk of forming nonspecific products [27]. To address this issue, probes are often incorporated into the LAMP system to enhance specificity and reduce the likelihood of nonspecific amplification [27]. Probes can be used to either monitor the amplification result or confirm the presence of specific target DNA. During the amplification process, probes bind to the target DNA region, allowing for the detection of successful amplification and ensuring the specificity of the target. For example, fluorescent probes enable real-time monitoring of the amplification process [29]. As the amplification product forms, the probe binds to the target DNA region, leading to an increase in fluorescence emission. This allows for real-time monitoring of the amplification process. Utilizing probes for real-time fluorescence reading in LAMP reactions enhances sensitivity, specificity, and accuracy and ensures compatibility with most standard isothermal amplification techniques [29]. LAMP assays that utilize probes not only facilitate the diagnosis of infectious diseases and pathogens detection but also enable the detection of pathogen strains or species with high phylogenic similarity within a single analysis [30,31]. Numerous studies have explored integrating different types of fluorescently labeled probes into the LAMP principle [27,28], including methods like the Detection of Amplification by Release of Quenching (DARQ) [29].

In recent years, efforts have been made to develop PoC tests for detecting MPXV. Aslan et al. developed a label-free digital detection platform that utilizes an Interferometric Reflectance Imaging Sensor to capture MPXV via an anti-A29 monoclonal antibody on Protein G spots with a limit-of-detection (LoD) value of 200 PFU/mL (~3.3 aM) [3]. Peng et al. designed a device that integrated Si-OH-magnetic-bead-based nucleic acid extraction to detect MPXV [32]. The colorimetric detection of the LAMP reaction was limited to 137 copies/mL [32]. Similarly, Chen et al. developed a LAMP-based detection reagent for Mpox utilizing a magnetic-bead-based nucleic acid extraction system with a sensitivity of 100 copies per μL [33]. Alternatively, Yu et al. developed a LAMP assay with primer sets designed to target regions in and around the *N4R* gene [34]. The results showed a detection limit of 2 × 100 DNA copies. Despite all these endeavors, the full potential of Mpox and Mpox clade detection remains underexplored. Several strategies can be leveraged to achieve higher sensitivity and improve feasibility. This is particularly crucial for clade discrimination, as the progression and associated disease severity for each clade are distinct.

In this study, we developed two new LAMP-based molecular assays to detect two different clades of MPXV. Clade-I contains *D13L*, *D14L*, *D15L*, *D16L*, *D17L*, and *D18L* genes, while in Clade-II, the *D18L* gene is positioned after the *D13L* gene due to deletion [35]. A region encompassing the *D14L*, *D15L*, *D16L*, and *D17L* genes is deleted in Clade-II (Clade IIa and IIb) but present in Clade-I (Clade Ia and Ib) [35,36,37]. This deletion serves as a key marker for distinguishing between the two clades, and our study aimed to detect this deletion. We first developed the LAMP-1 assay, which includes gene sequences present in Clade-I but absent in Clade-II. This assay has both fluorescence and colorimetric readout capabilities, making it suitable for PoC use. Secondly, we created a LAMP-2 assay specifically designed to target the deletion region in Clade-II gene sequences. In addition, a novel R-Duplex DARQ probe was developed for the LAMP-2 assay to ensure precise detection.

## 2. Methods

### 2.1. Target Site and DNA Template Preparation

A region spanning the *D14L*, *D15L*, *D16L*, and *D17L* genes in Clade-I is deleted in Clade-II, serving as a crucial marker for distinguishing between these two clades [35]. This deletion, a 1952 nt region from positions 18,953 nt to 20,905 nt in the MPXV Clade-I genome, is absent in Clade-II (Supplementary Figure S1 and Supplementary Table S1). Genomic sequence data of reference genomes of MPXV-Zaire-96-I-16 (GenBank: AF380138.1) for Clade-I and MPXV-M5312_HM12_Rivers (GenBank: MT903340.1) for Clade-II, obtained from the NCBI, were used to select the target gene regions. The 2451 bp gene region from the Clade-I genome, containing the *D13L*, *D14L*, *D15L*, *D16L*, *D17L*, and *D18L* genes, and a 500 bp gene region from the Clade-II genome, including *D13L* and *D18L* genes, were chosen for cloning. These gene regions were cloned into separate plasmids using the pUC57 vector. Plasmid maps were designed using the SnapGene 8.0.1 program to ensure the insertion of the target sequences into the multiple-cloning site (MCS). The plasmids, pUC57_Clade-I_, and pUC57_Clade-II_, with the target gene sequences cloned, were synthesized (GeneSuz, Istanbul, Turkey) and utilized as template DNA in LAMP assays. The stock plasmid constructs were measured with a NanoDrop (Thermo Scientific^TM^ NanoDrop^TM^ 2000, Waltham, MA, USA), diluted to 100 ng/μL with nuclease-free water, and prepared for use as template DNA.

### 2.2. Genetic Mapping and Primer Design for LAMP Assays

To design LAMP primers, the 2451 bp gene region containing the sequences of the *D13L*, *D14L*, *D15L*, *D16L*, *D17L*, and *D18L* genes was genetically mapped according to the Clade-I reference isolate (GenBank: AF380138.1). Based on the gene sequences of Clade-I, genetic maps containing the orthologous gene regions of the Clade-II reference isolate (GenBank: MT903340.1) and five viruses belonging to the *Orthopoxvirus* family were created and matched in Molecular Evolutionary Genetics Analysis Version 11 Software (Mega 11). The viruses with matched genetic maps are Vaccinia virus WR (Western Reserve) (GenBank: AY243312), Vaccinia virus Copenhagen (GenBank: M35027.1), Camelpox virus M-96 from Kazakhstan (GenBank: AF438165.1), Cowpox virus Brighton Red (AF482758.2), and Variola virus India-1967, ssp. major (GenBank: X69198.1) (Supplementary Table S1) (Supplementary Figures S1 and S2). Additionally, a phylogenetic tree containing the relevant viruses was created based on the Clade-I gene sequence (Supplementary Figure S2). Considering the similarities and differences, LAMP primer design was started.

The unique primer design principle of the LAMP method includes 2 outer primers (F3, B3), 2 inner primers (FIP = F1c + F2, BIP = B1c + B2), and 2 loop primers (LF, LB). LAMP assay primer designs were created using the Primer Explorer V5 program (Eiken Chemical Co., Tokyo, Japan). All LAMP assay primers were HPLC-purified and synthetically synthesized (Sentebiolab, Ankara, Turkey). For the LAMP-1 assay, LAMP primers were designed by targeting the *D14L* gene sequence on the Clade-I genome sequence. The purpose of using the LAMP-1 assay is to detect clades since the sequences of the primers and the targeted amplicon region are not present in Clade-II.

In the LAMP-2 assay, LAMP primers were designed by targeting the sequences in the 5′ and 3′ directions of the region where the deletion is located. The F2_LAMP-2_ primer was designed to cover the deletion region in both directions, 5′ and 3′. In the LAMP method, the F2_LAMP-2_ primer is a part of the FIP_LAMP-2_ primer and is one of the regions where it binds to the template nucleic acid sequence and starts amplification. Since there is no deletion in Clade-I, the FIP_LAMP-2_ primer cannot be bound, while Clade-II can also be bound by the FIP_LAMP-2_ primer. In addition, in the LAMP-2 assay, specific detection with the quencher probe principle was targeted by using the FIP_LAMP-2_ primer sequence as a template. Many LAMP primer sets were designed for both assays, and the most stable sets were selected. Sequences of stable LAMP primers designed for the clades are shown in Table 1.

### 2.3. Novel R-Duplex DARQ Probe Design

A duplex DARQ probe was designed to replace the FIP_LAMP-2_ primer in the LAMP reaction. This duplex structure consists of two nucleic acid strands, one with a 6-carboxyfluorescein (FAM) end and the other with a Black Hole Quencher 1 (BHQ1) end. The primary strand of the probe is designed according to the FIP_LAMP-2_ primer sequence and has FAM at the 5′ end (F1c_LAMP-2_ region). In addition, a GC-rich box was added between F1c_LAMP-2_ and F2_LAMP-2_ to facilitate the duplex formation of the two strands with each other at the LAMP reaction temperature. The secondary strand was designed to be the complement of the F1_LAMP-2_ sequence and the GC-rich box sequence. As it contains BHQ1 at the 3′ end of the second strand, it is able to quench the fluorescence signal as a result of the two strands forming a duplex structure during the reaction. Two different versions of the secondary strand were created, one ending with BHQ1 (DARQ-1) and the other ending with BHQ1-AG (DARQ-2) (Table 1). The designed duplex DARQ probe was synthesized synthetically (ProbeSynthesis Inc., Ankara, Turkey). While the standard duplex DARQ probe contains a quencher at the 5′ end of the FIP_LAMP-2_ primer, our new design contains FAM, which we called the reverse duplex DARQ probe (R-duplex DARQ).

### 2.4. LAMP Reactions

LAMP primers are designed to bind to specific DNA sequences (100% complementary) in the target region, as described in Section 2.2. The outer primers (F3 and B3) bind to the end regions of the target DNA, while the inner primers (FIP and BIP) bind to shorter internal sequences. This binding pattern only amplifies the target DNA region and prevents non-specific binding. In the LAMP reaction, the DNA polymerase enzyme, in addition to its amplification function, also ensures the opening of the double-stranded DNA, eliminating the need for a DNA denaturation step. This facilitates the sequence-specific binding of LAMP primers to the target region [27]. For each LAMP assay designed to detect clades, primer sets were tested to ensure adequate binding to the target region and amplification product formation. Each primer was dissolved in nuclease-free water into a 100 μM concentrated stock. As recommended in the LAMP protocol, primers were set in the reactions at concentrations of 2 μM of F3 and B3, 8 μM of LF and LB, and 16 μM of FIP and BIP. Mixes containing 6 primers from each LAMP primer set were created. Plasmid constructs took part in LAMP reactions as DNAs of a certain genotype. Concentrations of 25 ng per microliter of plasmid were prepared, and 1 μL was used in the reaction. For both the LAMP-1 and LAMP-2 assays, LAMP reaction was performed for fluorescence analysis. In addition, a colorimetric LAMP reaction was executed for the LAMP-1 assay.

In the fluorescence analysis, LavaLAMP^TM^ DNA Component Kit (GeneSuz, Istanbul, Turkey) was used chemically in the LAMP reactions and contained 2.5 μL of primer mix, 2.5 μL of 10× buffer, 1 μL of enzyme, 2.5 μL of Magnesium Sulfate (100 mM), 2 μL of dNTP mix (10 mM), and 1 μL of green dye (intercalating dye). The total reaction volume was increased to 25 μL with nuclease-free water. A negative control (NTC) containing non-template DNA reaction components was included simultaneously in the reactions. Additionally, positive control (PTC, LavaLAMP^TM^, GeneSuz, Istanbul, Turkey) reactions were performed. For each LAMP assay, one sample used pUC57_Clade-I_ plasmid DNA and another used pUC57_Clade-II_ plasmid DNA as template DNAs. LAMP reactions were carried out on a qPCR device (Roche, 480 LightCycler, Roche Diagnostics, Basel, Switzerland), and the reaction results were monitored via fluorescence. The reactions were designed to undergo 60 cycles, with fluorescence measurements made every 30 s. Each cycle was designed to be 30 s. The cycles were not designed for thermal cycling but fluorescence measurement. The aim was to create an amplification curve by taking fluorescence measurements in each cycle. Fluorescence reading was performed by selecting the FAM channel (480–520 wavelengths) on the qPCR device. The temperature range of the relevant kit is between 68 and 72 °C, and the reactions were carried out at 68 °C, 70 °C, and 72 °C to evaluate the optimum temperature value. Our criterion for the optimum temperature was the temperature that provided the highest amplification efficiency and fluorescence signal intensity in LAMP reactions. For this purpose, reactions were tested at different temperature ranges (e.g., 68–72 °C). The fluorescence signal intensity and amplification rate obtained at each temperature were evaluated, and the temperature that provided the highest signal and the shortest detection time was determined to be optimum. The optimum temperature value of our LAMP assay was found to be 68 °C.

Firstly, green dye (intercalating dye) was used in the LAMP-2 assay to detect the LAMP primers’ reactions. After confirming amplification, probe-based strategy was switched, and Novel R- Duplex DARQ probes were used instead of green dye.

In the LAMP reaction for colorimetric analysis, WarmStart^®^ Colorimetric LAMP 2× Master Mix (New England Biolabs, Ipswich, MA, USA) was utilized. Here, 1 μL of DNA (pUC57_Clade-I_ or pUC57_Clade-II_), 12.5 μL of WarmStart^®^ Colorimetric LAMP 2× Master Mix, and 2.5 μL of primer mix (LAMP-1 assay primers) were used in the reactions. Nuclease-free water was added in each reaction to make the total volume 25 μL. A negative control reaction without DNA was also performed simultaneously. LAMP reactions were carried out by using the PCR device (BIO-RAD, T100 PCR thermal cycler, Hercules, CA, USA) as a heater and incubating the mixture for 30 min at a constant temperature of 65 °C (the recommended temperature for the kit). Afterward, the reaction mixture was incubated at 4 °C degrees for 5 min to terminate the enzymatic activity. Since reading is performed according to a visible color change at the end of the reaction in colorimetric detection, no cycles were used for the reactions.

In this study, we used different types of LAMP result readout strategies to develop LAMP-1 and LAMP-2 assays. LAMP-1 assay offers the possibility of carrying out reactions in any heater device/block using only LAMP primers. Fluorescent or colorimetric detection can be selected for result readout based on user preference. This approach will allow the detection of Mpox clades at the PoC. In LAMP-2 assay, a probe-based approach was used after amplification of specific regions using LAMP primers to achieve a high level of sequence specificity.

### 2.5. Graphical User Interface (GUI)

A graphical user interface (GUI) was developed in the MATLAB environment to assist in measuring the intensities and similarity indexes of the collected samples relative to a controlled sample. The user can employ, with no prior knowledge of coding, the GUI application to visualize and quantify the given images. This not only facilitates result interpretation but also provides a platform on which to enter parameters in an efficient way. The user can upload an image from the library, which is displayed in the app. Then, the user must enter the number of samples that are being recorded in the image. Afterward, the user specifies a single diagonal corner of each visible sample (e.g., upper left and bottom right or upper right and bottom left), which contains the sample. The coordinates of the corner points are recorded within the app and processed to obtain a square-cropped area of the sample. Once the user clicks on the “Plot” button, the app extracts the grayscale image from square areas and plots the average intensities of the samples. Finally, the user is capable of extracting the intensities into a text file format.

### 2.6. LAMP Assay Labeled Novel R-Duplex DARQ Probe

In the probe-based analysis, LAMP reactions were created for 2 different R-Duplex DARQ probes that were newly designed using LAMP-2 assay primers. The DARQ probe structure designed consists of two complementary single DNA arms. Before the LAMP reaction, to obtain a duplex structure, the two synthesized arms were placed in an Eppendorf tube at equal concentrations and incubated at 95 °C for 5 min (BIO-RAD, T100 PCR thermal cycler, Hercules, CA, USA) and kept at room temperature for 30 min. The complementary regions of the arms were matched correctly to form a duplex structure. This preliminary step was applied separately for both designed probes. LavaLAMP^TM^ DNA Component Kit (GeneSuz, Istanbul, Turkey), which does not contain chemical intercalation dye, was used. A primer–probe mixture (for both R-Duplex DARQ probes) was prepared, containing 2 μM of F3_LAMP-2_ and B3_LAMP-2_, 8 μM of LF_LAMP-2_ and LB_LAMP-2_, 16 μM of BIP_LAMP-2_, 12 μM of FIP_LAMP-2_, and 4 μM of R-Duplex DARQ probe. The reaction mixture included 2.5 µL of primer-probe mixture, 2.5 µL of 10× buffer, 1 µL of enzyme, 2.5 µL of magnesium sulfate (100 mM), and 2 µL of dNTP mixture. The reaction volume was then brought to 25 µL by adding nuclease-free water. For each LAMP analysis, pUC57_Clade-I_ plasmid DNA was used in one sample, and pUC57_Clade-II_ plasmid DNA was used in the other. LAMP reactions were performed using the qPCR device (BIO-RAD, CFX Opus 96, Hercules, CA, USA) and monitored via fluorescence measurements with the FAM channel. Reactions were run in 60 cycles, and fluorescence measurements were obtained every 30 s. Since the optimum temperature value of the LAMP-2 assay with intercalation dye is 68 °C, the reactions were carried out at this temperature.

### 2.7. Sensitivity Analysis of LAMP Assays

The limit of detection (LoD) was determined using different initial DNA concentrations. DNA concentrations were converted to copy numbers using DNA molar mass and Avogadro’s number. The molecular weight of the plasmid DNA used in this study was calculated by considering the length of the DNA sequence and the average molecular weight of the base pairs. The reaction volume was 25 µL, and 1 µL of DNA sample was used for each reaction. Thus, the initial DNA amount per reaction was calculated in terms of copy numbers. The LoD was defined as the lowest DNA copy number at which a positive amplification signal could be obtained.

LAMP reactions were designed separately for the LAMP-1 and LAMP-2 assays at different DNA concentrations. Since the gene region specific to Clade-I was targeted in the LAMP-1 assay, pUC57_Clade-I_ plasmid DNA was used to analyze the LoD. The stock pUC57_Clade-I_ plasmid DNA concentration of 100 ng/μL was serially diluted 10-fold with nuclease-free water. In the LAMP-1 assay, two different LAMP kits are used for fluorescence and colorimetric signal generation. For this reason, reactions were performed separately for 10-fold serial dilutions of DNA (pUC57_Clade-I_) for both detection methods. LAMP-2 assay is designed to detect Clade-II by targeting the deletion region, and pUC57_Clade-II_ plasmid DNA was used in the sensitivity analysis. Probe-based LAMP reaction was performed with multiple serial dilutions of DNA. Sensitivity analysis was conducted separately for R-Duplex DARQ-1 and R-Duplex DARQ-2.

### 2.8. qPCR Reaction

qPCR reaction was performed for MPXV clade detection. Reactions were set up with Clade-I DNA, Clade-II DNA, and negative control (without DNA) samples using the LAMP-1 assay F3_LAMP-1_ (ACAATCTGACACGTGGGT) primer and LF_LAMP-1_ (CGTTGATTGGTAACTCTGGTGT) primer using the SYBR Green Quantitative RT-PCR Kit (Sigma-Aldrich, Merck KGaA, Darmstadt, Germany). Each reaction contained 5 μL of 2× master mix (SYBR Green Quantitative RT-PCR Kit), 0.5 μL of F3_LAMP-1_, 0.5 μL of LF_LAMP-1_, 1 μL of DNA, and 3 μL of nuclease-free water. Reactions were carried out using a QPCR device (Roche, 480 LightCycler) with the following cycling conditions: (1) initial denaturation (95 °C for 5 min), (2) 30 cycles of denaturation (95 °C for 45 s), annealing (65 °C for 45 s), and elongation (72 °C for 30 s) and (3) termination (40 °C for 5 min).

## 3. Results

**Figure 1:**
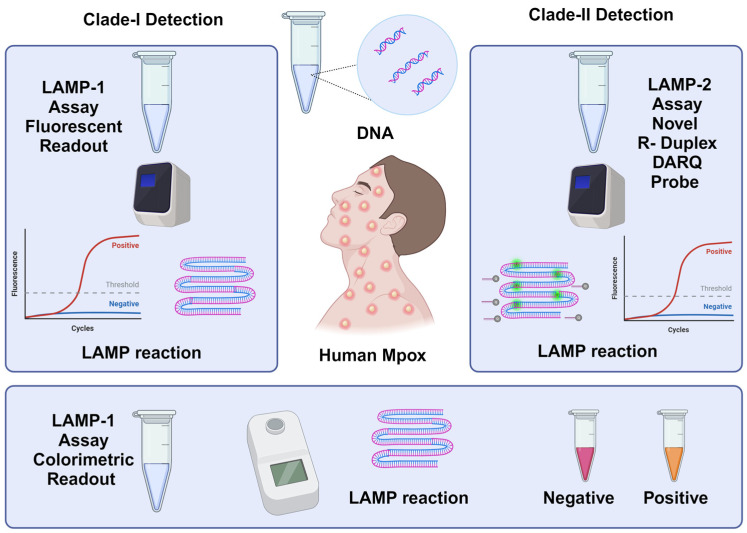
Three different molecular tests were developed using the LAMP method for MPXV Clade detection and are shown in the figure. Schematic diagram showing the workflow of LAMP-1 and LAMP-2 for Clade-I and Clade-II detection. LAMP-1 assay was used to detect Clade-I. The amplification was verified based on fluorescent emission and a colorimetric change from pink to yellow. LAMP-2 assay was used to detect Clade-II. The probe-based LAMP method can achieve fluorescence detection by using the R-Duplex DARQ probe for Clade-II detection.

In this study, three distinct assays were successfully developed for the detection of Mpox clades (Figure 1). The LAMP-1 assay, which includes both fluorescence and colorimetric tests, was designed to identify Clade-I. The fluorescence test of the LAMP-1 assay exhibited high sensitivity, while the colorimetric test provided a visible readout suitable for PoC applications. For Clade-II detection, the LAMP-2 assay was employed, utilizing a probe-based method to ensure precise identification of this clade. The results demonstrated that each assay effectively distinguished between the Mpox clades, showcasing the robustness and applicability of the developed tests.

### 3.1. Genetic Mapping and Phylogenetic Analysis for Primer Design in LAMP Assays

Phylogenetic analysis was conducted to elucidate the genetic relationships within the *Poxviridae* family *(Orthopoxvirus* genus), particularly focusing on MPXV Clade-I. The analysis traced the evolutionary paths and relationships among various *Poxviridae* species, with a total of seven virus species being represented in the phylogenetic tree (Supplementary Figure S2). Vaccinia virus WR and Vaccinia virus Copenhagen showed the closest relatedness, with a minimal genetic distance of 0.001. These two types were strongly supported, with a bootstrap value of 88, indicating the robust reliability of this branching. In comparison to MPXV Clade-I, Vaccinia virus species are separated by a genetic distance of 0.026, suggesting that MPXV Clade-I has a more distant evolutionary relationship with Vaccinia viruses. MPXV Clade-I and the Cowpox virus (Brighton Red strain) are closely related, with a genetic distance of 0.004, indicating that the Cowpox virus is a very close relative of MPXV Clade-I. In contrast, the Camelpox virus M-96, from Kazakhstan, branches from MPXV at a genetic distance of 0.016, suggesting a more distant evolutionary relationship and a more remote common ancestor. The Variola virus exhibits the greatest genetic distance from both MPXV Clades-I and Clade-II, with a separation of 0.023. This considerable distance indicates that the Variola virus has a much more distant evolutionary relationship than the other *Poxviridaes,* reflecting significant divergence.

In addition, the primer sequences of the LAMP-1 and LAMP-2 assays, when compared with genetic maps, reveal nucleotide differences and small deletion regions in relation to the other five viruses (Supplementary Figure S1). Both the LAMP-1 and the LAMP-2 assays were designed to selectively target gene sequences that are distinct from those of five other virus species. Additionally, the targeted deletion region was not present in the genetic maps of these other five viruses. As a result, the FIP primer region of the LAMP-2 assay, which employs the R-Duplex DARQ probe, demonstrates a high level of selectivity (Supplementary Figure S1). The R-Duplex DARQ probe is a highly specific detection tool capable of distinguishing Clade-II from other members of the *Orthopoxvirus* family.

### 3.2. Fluorescent Readout of the LAMP-1 Assay

**Figure 2:**
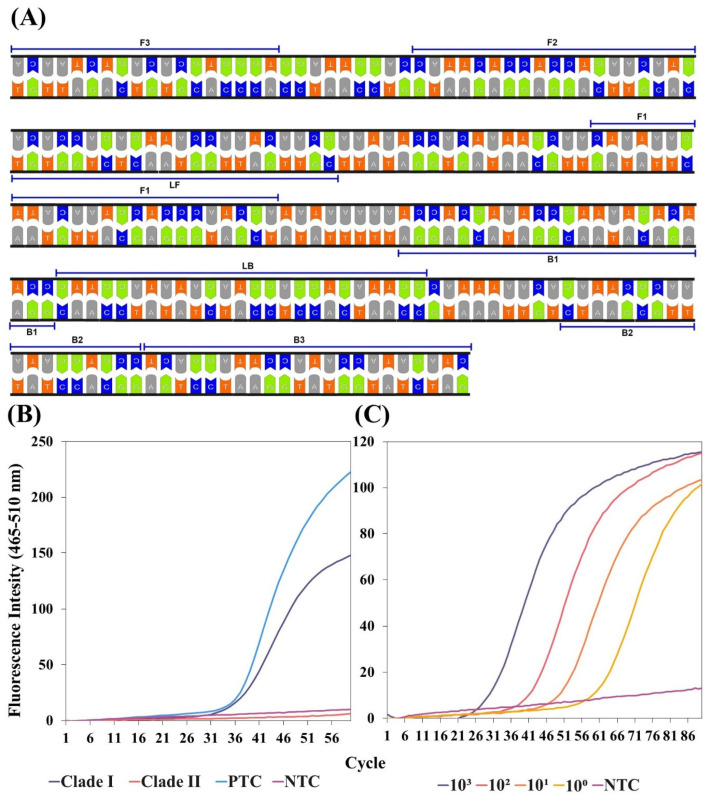
A scheme depicting primers and target gene for Clade-I detection, specificity, and sensitivity results. (**A**) Location of LAMP-1 assay primers on *D14L* sequences. (**B**) LAMP-1 fluorescence reading results for clade detection. The successful amplification of Clade-I within approximately 18 min with no Clade-II amplification indicates high specificity of the assay. (**C**) LoD analysis of template DNA with 10-fold serial dilution samples for LAMP-1 assay. The sensitivity of the tests is 10^3^ copies. PTC: positive control. NTC: negative control.

The objective was to differentiate between the two MPXV clades by targeting Clade-I detection with the LAMP-1 assay. Notable genetic differences between Clade-I and Clade-II were identified as potential indicators. To exploit these differences, the *D14L* gene region, which is present in Clade-I and absent in Clade-II, was selected for this assay. Six LAMP primers were designed to target the *D14L* gene (Figure 2A). The LAMP reaction was carried out at a constant temperature of 68 °C for 30 min, without thermal cycling, and an intercalation dye for fluorescence detection was employed. Under these conditions, stable and reliable amplification was achieved in an isothermal environment.

The LAMP reaction was carried out using Clade-I and Clade-II plasmid DNAs (pUC57_Clade-I_ and pUC57_Clade-II_), along with negative and positive controls. For Clade-I DNA (pUC57_Clade-I_), amplification curves started to appear at a Cp value of 34.5, with each cycle lasting 30 s (Figure 2B). Fluorescent detection confirmed that the LAMP-1 assay primers successfully amplified the Clade-I-specific *D14L* gene region. Clade-I presence was detected within approximately 18 min, demonstrating the efficacy of the LAMP-1 assay.

In contrast, no amplification was observed with Clade-II DNA (pUC57_Clade-II_) (Figure 2B). This absence of amplification confirms that the *D14L* gene region is not present in Clade-II, proving that this method does not detect Clade-II. The lack of amplification of Clade-II DNA (pUC57_Clade-II_) highlights the high specificity of the primers and the remarkable sensitivity of this method. These results show that the LAMP-1 assay is an effective and reliable molecular diagnostic tool for distinguishing between MPXV clades using a fluorescent readout.

### 3.3. Colorimetric Readout of the LAMP-1 Assay

**Figure 3:**
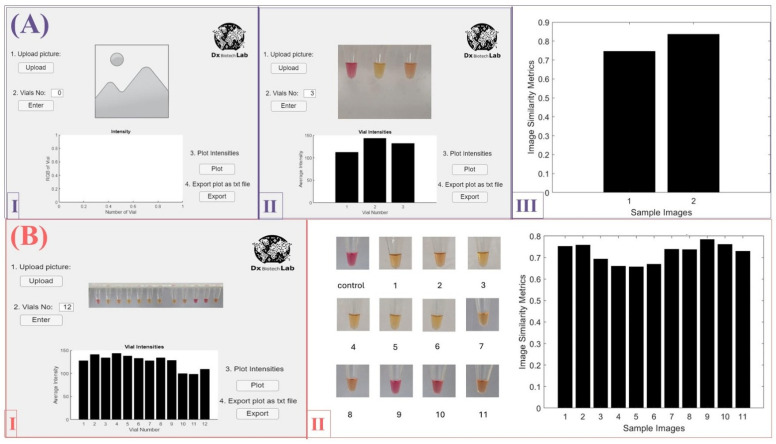
LAMP-1 assay colorimetric reading results. (**A**) Screenshot of the developed MATLAB-based GUI. The interface displays sections for image upload, vial count entry, and sample selection through diagonal corner coordinates. The GUI measures average sample intensities and displays them in a bar plot, with options to export the bar plot values as a text file. (**B**) Screenshot of the MATLAB-based GUI displaying an image with twelve samples, including the negative control tube (Sample 1), with specified corners and the resulting bar plot of average sample intensities. The GUI accurately captures the relative difference in sample intensities, highlighting samples 10 and 11 in comparison to the control sample, which signifies the functionality of the app.

To enhance Clade-I detection with the LAMP-1 assay, a visible colorimetric readout was employed alongside the fluorescent signal as an additional molecular diagnostic tool. Our aim was to elevate the PoC utility of the LAMP-1 assay, making it easier to use without requiring specialized equipment. Detection was based on color changes resulting from pH alterations associated with the amplification process. A pH change leads to a color shift from pink to yellow. Therefore, pink indicates the absence of amplification, signifying a negative result, while yellow indicates the presence of amplification, signifying a positive result. After incubation at 65 °C for 30 min, the results of the LAMP-1 assay with the negative control and Clade-II DNA (pUC57_Clade-II_) appeared pink when viewed with the naked eye. In contrast, the assay sample containing Clade-I DNA (pUC57_Clade-I_) turned yellow, indicating a positive result. This result showed that no amplification occurred in the negative control and Clade-II sample, while amplification was present in the Clade-I sample (Figure 3A). Therefore, the LAMP-1 assay effectively detects Clade-I using a colorimetric readout visible to the naked eye and successfully differentiates between the two MPXV clades.

In our first test for measuring intensity, an image with three samples was uploaded. The GUI prompted us to identify the corners of each sample. After this step, we were able to obtain and visualize the intensities in a bar plot, as shown in Figure 3A. The intensity levels varied among the three samples. The first sample, which served as the negative control, showed the lowest intensity. Sample 2 exhibited the highest intensity, while sample 3 and the control had relatively lower, but still distinct, intensities. These variations highlight differences in the sample composition and contribute to the observed results.

### 3.4. LAMP-1 Assay LoD Analysis

Sensitivity analyses were performed in separate reactions for both fluorescence and colorimetric readings. In these reactions, an LoD value of 10^3^ copies was defined as the DNA amplification capacity of the LAMP-1 assay in fluorescent analysis (Figure 2C). Amplification of 100 ng/μL, 10 ng/μL, 1 ng/μL, and 0.1 ng/μL was observed in Clade-I DNA (pUC57_Clade-I_) samples using 1 μL of 10-fold serial dilution. It has thus been proven that the LAMP-1 assay is sensitive enough to easily detect 0.1 ng/μL of DNA.

It was found that the LAMP-1 assay is capable of amplifying Clade-I DNA (pUC57_Clade-I_) with an LoD of 10^7^ copies while offering colorimetric detection visible to the naked eye (Figure 3B). As a result of the reactions performed with DNA samples at different concentrations created by 10-fold serial dilution of 100 ng/μL DNA, it was demonstrated that the LAMP-1 assay was able to detect 0.01 pg/μL of DNA in colorimetric analysis.

The LoD concentrations of 0.1 ng/μL (fluorescence readout) and 0.01 pg/μL (colorimetric readout) were determined using 10-fold serial dilutions of plasmid DNA at an initial concentration of 100 ng/μL. The molecular weight of the plasmid DNA used in these experiments was 3 × 10^6^ g/mol, and through calculations made using Avogadro’s number, it was determined that the solution with a concentration of 100 ng/μL contained approximately 2.007 × 10^7^ copies/μL. The DNA concentrations and corresponding copy numbers obtained after 10-fold serial dilutions are as follows: 0.1 ng/μL corresponds to approximately 10^3^ copies (Figure 2C), and 0.01 ng/μL corresponds to approximately 10^7^ copies (Figure 3B).

In the sensitivity analysis for the colorimetric readout, we again uploaded an image in the GUI but this time with twelve samples, with the first sample serving as the negative control. The GUI processed the image and was asked to plot the average intensity, showing variation among the samples (Figure 3B). Samples 2, 3, 4, 5, 6, 7, 8, and 9 exhibited the highest intensities, while samples 10, 11, and 12 had the lowest. The negative control, sample 1, provided a baseline reference that enabled comparisons of the intensity levels among other samples, and all other samples showed higher intensities relative to the control. In general, to mitigate possible errors in the intensity readout, we defined a standard procedure for determining the region of interest across all vials. Since the region of interest is selected by the user, regardless of the diameter of the vial, the algorithm considers the normalized intensity of that selected region in taking the average intensity of the individual pixels. The MATLAB (MathWorks Inc., Natick, MA, USA, R2021b) tool indeed measures the intensity of the vials as a quantitative representation of their colorimetric response. The intensity readout provides a direct correlation with the concentration of the target analyte; a concentrated analyte has a higher intensity index compared to a less concentrated analyte. Color measurements could offer additional information; however, intensity measurements were sufficient for this work.

### 3.5. qPCR Analysis for Clade-I Detection (Targeted in LAMP-1 Assay)

Clade detection was also successfully achieved with the qPCR reaction using the two primers (F3 and LF) of the LAMP-1 assay. Since the regions of all primers in the LAMP-1 assay were found in Clade-I but not in Clade-II as a result of the deletion, detection was also achieved via qPCR. While there was an amplification curve in Clade-I (pUC57_Clade-I_), there was no amplification curve in Clade-II (pUC57_Clade-II_). In qPCR, the results were monitored with a reaction that included a 70 min thermal cycle, while in the LAMP reaction, the results were monitored for 30 min. The results obtained showed that amplification occurred at a Ct value of 18.6 for Clade-I, and there was no amplification for Clade-II. These findings emphasize the specificity and sensitivity of the experiments. These results support the reliability of the Clade-I detection ability of the LAMP-1 assay.

### 3.6. LAMP-2 Assay Primer Optimization

**Figure 4:**
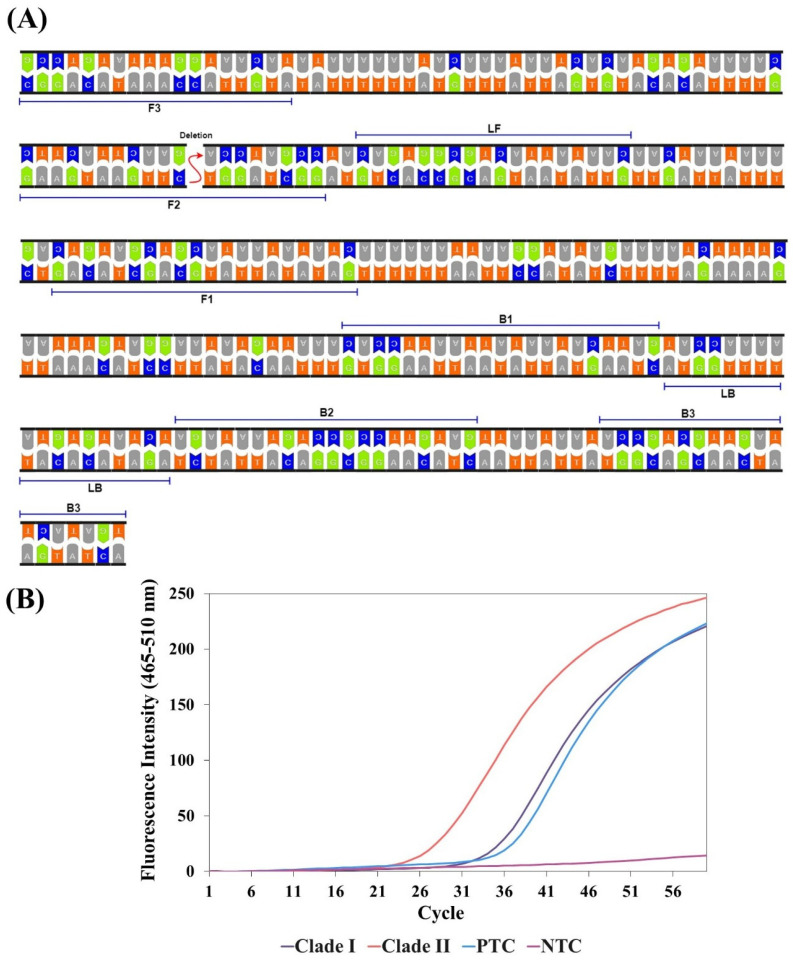
A scheme depicting primers and target gene for Clade-II detection and specificity results. (**A**) Location of LAMP-2 assay primers on target gene sequences. (**B**) LAMP-2 fluorescence reading results for Clade detection. The higher fluorescence intensity and earlier amplification of Clade-II indicate the successful detection of Clade-II. PTC: positive control. NTC: negative control.

The LAMP-2 assay focuses on the detection of Clade-II. The region where the deletion was located was matched with the F2_LAMP-2_ region of the FIP_LAMP-2_ primer. The probe designed for this region was employed to enable the detection of Clade-II (Figure 4A). We expected that all primers of the LAMP-2 assay, except the FIP_LAMP-2_ primer, would also match the gene sequences of Clade-I. Only the FIP_LAMP-2_ primer region would not match. Therefore, before the probe experiment, an intercalation dye without a probe was sampled on the LAMP-2 to prove our assumption. At the same time, the optimum working conditions of the primers of the LAMP-2 assay were revealed. The Clade-II (pUC57_Clade-II_) sample started amplification at 25.4 cp, while the Clade-I (pUC57_Clade-II_) sample started amplification at 35.6 cp (Figure 4B). Clade-I experiments demonstrated that the FIP_LAMP-2_ primer cannot bind while other primers bind and produce amplification products. Failure to bind the FIP_LAMP-2_ primer resulted in delayed amplification. Therefore, this was an indication that a probe corresponding to the FIP_LAMP-2_ primer would allow detection. It was also observed that the LAMP-2 assay primers worked optimally at a constant temperature of 68 °C.

### 3.7. Fluorescence Detection with Probe-Based LAMP-2 Assay

**Figure 5:**
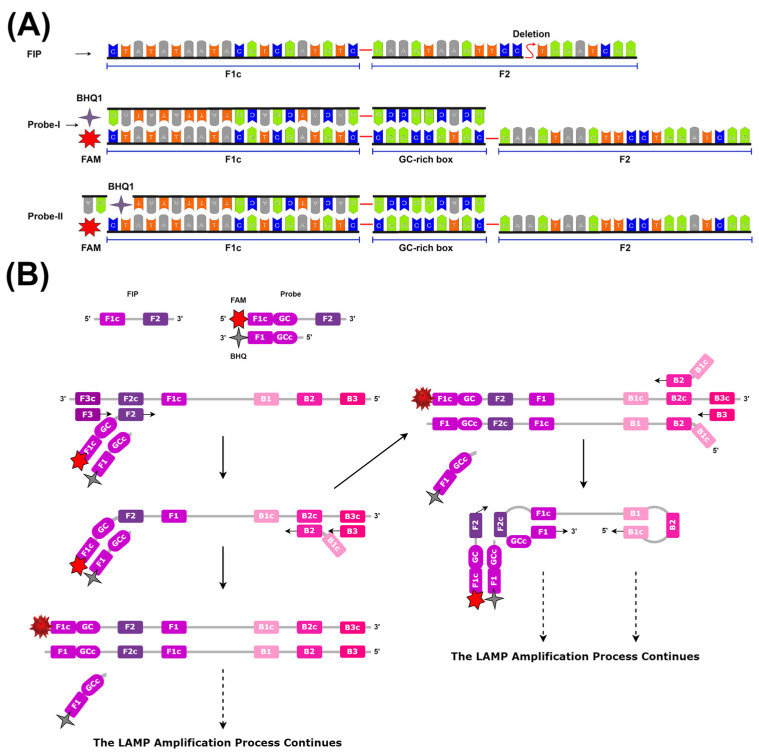
A schematic illustration of the structure and working principle of the fluorescent probe designed for Clade-II. (**A**) R-Duplex DARQ probe structure. (**B**) Working principle of R-Duplex DARQ probe in the LAMP reaction.

**Figure 6:**
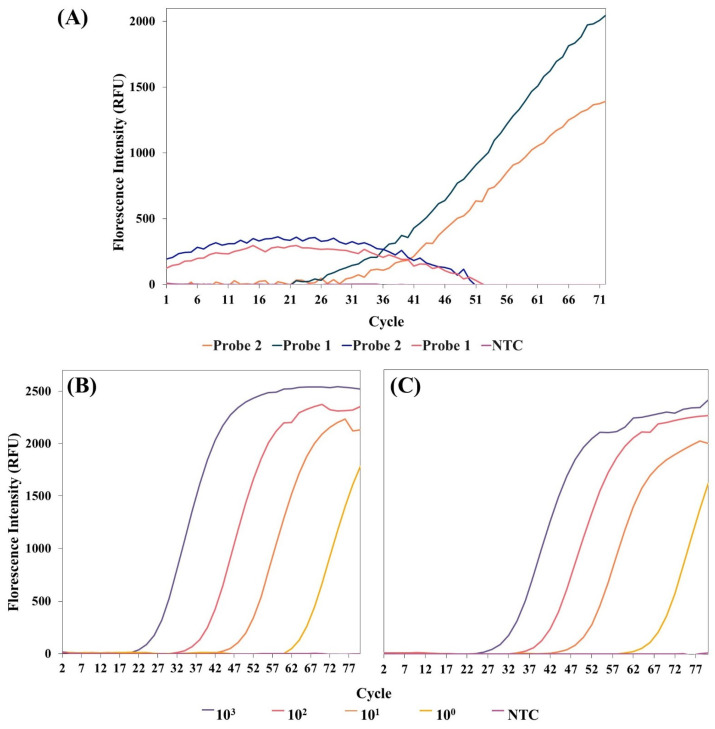
(**A**) Fluorescence readout of LAMP assay with new R- Duplex DARQ probes: In reactions performed with Clade-I DNA, no fluorescent signal was observed when using the R-Duplex DARQ-1 probe (red line) or the R-Duplex DARQ-2 probe (blue line). In contrast, in LAMP reactions performed with Clade-II DNA, fluorescent signal was detected using both the R-Duplex DARQ-1 probe (black line) and the R-Duplex DARQ-2 probe (orange line). This indicates that the probe-based LAMP-2 assay successfully achieved amplification in Clade-II DNA and enabled fluorescence detection. (**B**) LoD analysis of R- Duplex DARQ-1 probe. (**C**) LoD analysis of R- Duplex DARQ-2 probe. Both probes showed a sensitivity value of 10^3^ copies. NTC: negative control.

The FAM arm was added to the amplification product with a duplex DARQ probe structure, separating the quencher arm and irradiating it to detect Clade-II DNA (Figure 5A,B). While both R-Duplex DARQ-1 and R-Duplex DARQ-2 probes produced fluorescence in the FAM reading range in the Clade-II (pUC57_Clade-II_) sample, no fluorescence was detected in the Clade-I (pUC57_Clade-I_) samples (Figure 6A). Clade-II detection was achieved with both probes. Thanks to the GC-rich box added to the sequences of both probes, fluorescence was successfully achieved in the reactions at 68 °C, which is the optimum temperature value for the LAMP reaction (Figure 5A). The GC-rich box increases the melting temperature (Tm) of the probe, allowing it to remain hybridized to the target site and maintain its stability under reaction conditions at 68 °C. This stability reduces the probability of the dissociation of the probe from the target DNA and increases the reliability of fluorescence detection. In addition, the high GC content prevents secondary structure formation, resulting in a stronger and more consistent fluorescence signal. When both probes were compared, the R-Duplex DARQ-1 probe started to fluoresce in a shorter time than the R-Duplex DARQ-2 and produced a higher fluorescent signal at the same time (Figure 6A). Clade-II can be used as a molecular diagnostic tool as it provides clear selectivity in Mpox clade detection.

### 3.8. Probe-Based LAMP-2 Assay LoD Analysis

Sensitivity analysis was performed using Clade-II DNA (pUC57_Clade-II_) samples prepared separately in 10-fold serial dilutions in both R-Duplex DARQ probes. It was observed that both probes were sensitive enough to detect 0.1 ng/μL of DNA. Additionally, LoD values of 10^3^ copies were identified in the two probes (Figure 6B,C). However, the R-Duplex DARQ-1 probe started to produce fluorescence in the FAM range in a shorter time than R-Duplex DARQ-2 in all 10-fold serial dilution samples and gave a higher level of fluorescence in an equal amount of time. Both R-Duplex DARQ probes are sensitive enough to detect Mpox-Clade-II, and R-Duplex DARQ-1 has been shown to be preferable for use due to its greater fluorescence intensity.

## 4. Discussion

The most recent Mpox outbreak was declared a public health emergency of international concern by the WHO on 14 August 2024 in response to the rapid and extensive spread of Clade-I, which has raised concerns about its potential global impact. Mpox has attracted global attention following its incidence in non-endemic countries and its rapid spread globally in 2022 [15]. As a result of the global burden of the COVID-19 pandemic, the development of tools and strategies that can be employed rapidly and effectively when the next epidemic or pandemic occurs has attracted global attention. The first step in achieving control over epidemics and pandemics is the accurate diagnosis of diseases. Therefore, PoC diagnostic tests should be developed specifically for MPXV [38]. The two clades of MPXV show genetic, epidemiological, and clinical differences. The accurate identification of clades is important not only for predicting clinical outcomes but also allowing for the epidemiological mapping of disease spread. Besides showing many similarities among clades, MPXV also shows clinical and genetic sequence similarities with other viruses from the same family. Thus, it is imperative to also differentiate MPXV from other *Poxviridaes.* To this end, LAMP-based strategies have been employed to detect MPXV. It has been demonstrated that LAMP assays targeting *A27L-1* and *F3L-1* regions can detect MPXV with a higher sensitivity than the gold-standard PCR [39]. Similarly, portable LAMP-based assays with a visual detection limit of 137 copies/mL have been developed [40].

Here, we aimed to (1) differentiate MPXV from other *Poxviridaes* and (2) develop assays that can accurately and sensitively distinguish between MPXV Clades I and II. During the selection of the target gene region, we performed a phylogenetic analysis by matching the genetic map with the orthologous gene regions of four different viruses from the same family. Our genetic map matching and phylogenetic analysis data revealed in detail the evolutionary relationships of *Poxviridae* and the genetic differences of the target regions we identified for the LAMP assays. In particular, a clear separation of MPXV Clade-I and Clade-II in the phylogenetic tree and other *Poxviridaes* species showing great genetic distance will enable molecular identification that can be used to discriminate between these species. In this study, we focused on the detection of Clade-I and Clade-II of MPXV. We developed two different novel assays for the LAMP method, which is convenient for PoC settings. In our LAMP assays, we targeted a single deletion site that could clearly separate the two clades. Three different diagnostic tools were developed using three different techniques of LAMP technology. While the LAMP-1 assay allows monitoring with a fluorescent signal and colorimetric signal, the LAMP-2 assay allows fluorescence monitoring with a probe-based technique.

Using the LAMP-1 assay, Clade-I was detected in 30 min, allowing both fluorescent and colorimetric detection according to the user’s preference. Clade-II was detected using the LAMP-2 assay by targeting the gene sequence wherein the deletion was present. Additionally, Clade-I was detected via the LAMP-1 assay by targeting the gene region without deleting it. Using the LAMP-1 assay, Clade-I was detected in 30 min, and we equipped the assay with both fluorescent and colorimetric detection capabilities to offer flexibility in result interpretation according to the user’s preference. We were able to detect Clade-II in 40 min with the LAMP-2 assay and our newly developed R-Duplex DARQ probe. A few studies aimed to detect clades by targeting one or a few nucleotide changes using the qPCR method [16,32]. In one study, clade determination was made based on the difference in the 453rd nucleotide residue (a single-nucleotide change) in the *ATI* gene [32]. According to SNP differences, Clade-I detection was achieved with the *G2R_WA* gene, and Clade-II detection was achieved with the *C3L* gene via probe-based qPCR [16]. These studies reported LoD values of 8.2 and 40.4 copies, respectively. In our study, the LoD values were determined to be 10^3^ and 10^7^ copies in the fluorescence and colorimetric LAMP-1 assays, respectively, and 10^3^ copies in the LAMP-2 assay. A very high number of DNA copies was obtained in a shorter time compared to qPCR. In addition, our LAMP-1 and LAMP-2 assays offer a significant advantage compared to qPCR as they do not require complicated equipment or a strong laboratory infrastructure. Although the LAMP-1 and LAMP-2 assays differ in terms of the gene regions they target, they exhibit higher specificity than tests focusing on single-nucleotide changes. The results of this study show that the LAMP method can be successfully applied not only in the laboratory environment but also in field conditions. In addition, this assay is suitable for use in field conditions and can easily be integrated with portable devices. Moreover, the LAMP-1 and LAMP-2 assays have unique advantages over qPCR, particularly in real-world scenarios. Unlike qPCR and PCR, which traditionally demand expensive instrumentation and specialized laboratory settings [34,41,42], our LAMP assays offer a portable and cost-effective solution while maintaining high specificity and sensitivity. These features make LAMP-based assays ideal for PoC applications, especially in resource-limited settings. Additionally, their ability to amplify DNA in a shorter timeframe than qPCR underscores their potential for rapid and accurate diagnostics in clinical and field conditions. Thus, they have great potential for rapid diagnosis in emergencies. The LAMP assays developed in this study were designed to operate at the highest efficiency at a constant and optimal temperature. Under conditions other than the optimal temperature, the reaction kinetics may slow down and amplification may fail or be delayed. When a constant temperature is not provided, inconsistencies in primer binding and enzyme activity may occur, especially during rapid temperature changes. This may lead to the lack of formation of an amplification curve or false-negative results [26]. However, the LAMP kits used are optimized to tolerate a certain temperature range (e.g., 65–72 °C). It has been reported that devices with low-temperature fluctuations can make it difficult to detect low levels of DNA [43,44]. In addition, it has been stated that the optimum temperature range for LAMP reactions is generally 65–72 °C. [45] Therefore, it is important to provide constant and appropriate temperature conditions to acquire accurate results.

In a few studies, the Duplex DARQ probe was used in combination with LAMP technology [29,46]. For example, in one study, the FAM tail was released, and the quencher tail was assimilated into the amplification product. Here, we created a novel design by making a difference in the classical Duplex DARQ probe principle. Assimilated probe logic was used. The assimilated probe also assimilates into the amplification product during FAM fluorescent tail amplification, and the fluorescent signal increases as the amplification product increases [47,48]. Based on this principle, the FAM tail will assimilate into the probe, and the quencher tail will be released during amplification. Since the arm that simulates the classical Duplex DARQ probe principle has changed, it is called the reverse Duplex DARQ probe (R-Duplex DARQ probe). During the LAMP reaction, DNA synthesis proceeds by annealing the F2_LAMP-2_ primer region sequences on the FAM end-mounted arm of the R-Duplex DARQ probe to the template DNA. When BIP_LAMP-2_ or B3_LAMP-2_ primers backward anneal to the newly synthesized DNA strand and advances DNA synthesis, the quenching end arm of the R-Duplex DARQ probe is released. Thus, the FAM arm of the R-Duplex DARQ probe (containing FIP_LAMP-2_ sequences) will be assimilated into the amplification product, and fluorescence will occur in the amplification product. Our R-Duplex DARQ probe reacted successfully in the LAMP-2 assay and was monitored fluorescently. Clade-II detection was achieved. In Clade-I, no fluorescent signal was observed in the R-Duplex-DARQ-probe-based LAMP reaction. This explains why the FIP_LAMP-2_ primer could not bind to the target and did not participate in the amplification, because the long arm of the R-Duplex DARQ probe structure contains the FIP_LAMP-2_ primer sequence. Moreover, the absence of a signal in the probe reaction indicates that the probe did not bind. On the other hand, the absence of any low-level fluorescent signal indicates that no cross-reaction was possible. It is thought that by assimilating the R-Duplex DARQ probe into the amplification product of the FAM arm, the amount of FAM signal and the amount of amplification product can be monitored as well as detected. Signal amplification and real-time quantification can allow significant advancements in Mpox diagnosis. This dual capability not only enhances the detection process but also provides valuable quantitative insights into the amplification dynamics. The R-Duplex DARQ probe’s compatibility with LAMP technology suggests a promising avenue for the development of quantitative LAMP assays. By refining this approach, future advancements could enable highly precise measurement of target DNA concentrations, facilitating applications in clinical diagnostics, pathogen load quantification, and other scenarios where accurate quantification is critical. This integration could further enhance LAMP’s utility [32,49], bridging the gap between its qualitative strengths and the quantitative capabilities traditionally associated with qPCR. We foresee that quantitative LAMP assays can be developed in the future by using the R-Duplex DARQ probe principle along with LAMP technology.

The findings of this study reveal that the LAMP method is effective in detecting MPXV Clade-I and Clade-II rapidly and accurately. This method has a great advantage in terms of its applicability and practicality, especially in field conditions. It has been shown that rapid and reliable results can be obtained with the LAMP method in cases requiring high sensitivity and specificity. The ability to distinguish Clade-I and Clade-II also provides a great advantage in terms of epidemiological studies and epidemic management.

In conclusion, this study demonstrates that the LAMP method can be used as an effective molecular diagnostic tool for distinguishing between different clades of MPXV. The sensitivity and specificity of this method can be further increased with future studies, and thus LAMP-based strategies may find wider application in the detection of viral pathogens.

## 5. Conclusions

In summary, we have developed two robust LAMP assays for the detection of MPXV Clade-I and Clade-II, serving as practical and efficient diagnostic tools. The LAMP-1 assay provides dual readout options—fluorescence with a sensitivity of 10^3^ copies and colorimetric detection with a sensitivity of 10^7^ copies—demonstrating high sensitivity, rendering this assay applicable for various settings, including lower-resource areas where laboratory infrastructure may be limited. The LAMP-2 assay incorporates the novel R-Duplex DARQ probe, enabling fluorescence-based detection with a sensitivity of 10^3^ copies while also laying the foundation for future quantitative LAMP technologies. Both assays outperform qPCR in terms of speed and simplicity, eliminating the need for complex equipment or extensive laboratory infrastructure. In addition, by offering highly precise and accurate quantification, these strategies can be used in the development of other diagnostic tools. Clinical validation on a large number of clinical samples is required to further evaluate the use of these platforms in real-life settings. In addition, while these assays have great potential to enable the PoC diagnosis of Mpox clades, it is imperative to determine the conditions in which reagents remain stable. A lack of reagent stability under suboptimal environmental conditions (i.e., during transportation) can jeopardize the use of these assays in low-resource settings. If these limitations are overcome, the assays can facilitate rapid PoC diagnosis. Such advancements can allow for the fabrication of specific, sensitive, and accessible diagnostic platforms, which would prove particularly valuable for PoC applications and resource-limited environments.

## Figures and Tables

**Table 1:** LAMP assays primers and R-Duplex DARQ probe nucleotide sequences and R-Duplex DARQ probe nucleotide sequences.

LAMP Primers and Probes	Nucleotide Sequences 5′ → 3′
**F3_LAMP-1_**	ACAATCTGACACGTGGGT
**F2_LAMP-1_**	CATTCTCCTCCTGAACACATG
**F1c_LAMP-1_**	TCGATGGGAGCATTGTAACTTATAG
**B1c_LAMP-1_**	TCCTCGTATCCGTTATGTCTTCC
**B2_LAMP-1_**	GGCACCTATTTGCGAATC
**B3_LAMP-1_**	GATCTATGGTATGGAATCCTGA
**FIP_LAMP-1_**	TCGATGGGAGCATTGTAACTTATAG-CATTCTCCTCCTGAACACATG
**BIP_LAMP-1_**	TCCTCGTATCCGTTATGTCTTCC-GGCACCTATTTGCGAATC
**LF_LAMP-1_**	CGTTGATTGGTAACTCTGGTGT
**LB_LAMP-1_**	GTTGGATATAGATGGAGGTGATTGG
**F3_LAMP-2_**	CGGACATAAACCATTGTA
**F2_LAMP-2_**	GAAGTAAGTTCCTGGATCGG
**F1c_LAMP-2_**	CTATATAATACGTCGATGTC
**B1c_LAMP-2_**	GTGGAATTAATATTATGAATC
**B2_LAMP-2_**	GATGTTCCGCCTGTAATAGA
**B3_LAMP-2_**	TGATACTAGTTGCTGCCA
**FIP_LAMP-2_**	CTATATAATACGTCGATGTC-GAAGTAAGTTCCTGGATCGG
**BIP_LAMP-2_**	GTGGAATTAATATTATGAATC-GATGTTCCGCCTGTAATAGA
**LF_LAMP-2_**	CAATATTACTGCGGTGAC
**LB_LAMP-2_**	ATGGTTTTTACACATAGA
**R-Duplex DARQ-1 probe**	**FAM**-CTATATAATACGTCGATGTCCGGCCGTGCGAAGTAAGTTCCTGGATCGG GCACGGCCGGACATCGACGTATTATATAG-**BHQ1**
**R-Duplex DARQ-2 probe**	**FAM**-CTATATAATACGTCGATGTCCGGCCGTGCGAAGTAAGTTCCTGGATCGG GCACGGCCGGACATCGACGTATTATAT-**BHQ1**-AG

## Data Availability

Data are contained within the article.

## Supplementary Materials

The following supporting information can be downloaded at https://www.mdpi.com/article/10.3390/bios15010023/s1. Figure S1: Genetic map matching with MPXV-Clades and *Poxviridaes* family members and primer locations of LAMP assays on the map. The deletion found in Clade-II is not found in other *Poxviridae* family members. The LAMP-2 assay (R-Duplex DARQ-2 probe) can distinguish Clade-II from both Clade-I and *Poxviridae* family members (white-marked regions indicate deletions). All primers for the LAMP-1 assay were selected from the region present in the Clade-I genome sequence but deleted in Clade-II. LAMP-1 assay primer regions show nucleotide differences in different regions from *Poxviridae* family members. Figure S2: Phylogenetic tree represents the evolutionary relationships of Monkeypox Clade-I with other *Orthopoxvirus* species. Vaccinia viruses and Cowpox virus are relatively close relatives of MPXV Clade-I, split by genetic distances of 0.026 and 0.004, respectively. The genetic distance between MPXV Clade-II and Clade-I is 0.008. MPXV Clade-I separates Camelpox and Variola viruses by a genetic distance of 0.016 and 0.023, respectively, indicating more distantly related relationships. Table S1: Orthologous gene region mapping between MPXV-Clades and *Orthopoxvirus* family members and nucleotide locations of the region used in the phylogenic analysis.
