# Supplementary material for: Development and Validation of LAMP Assays for Distinguishing MPXV Clades with Fluorescent and Colorimetric Readouts
Source: Biosensors (Basel). 2025 Jan 6;15(1):23. doi: 10.3390/bios15010023 (PMC11764415; doi:10.3390/bios15010023)
Supplement: Supplementary file 1 [file biosensors-15-00023-s001.zip › biosensors-3379694-supplementary.pdf]

*Supplementary Materials*

# Development and Validation of LAMP Assays for Distinguishing MPXV Clades with Fluorescent and Colorimetric Readouts

**Nazente Atceken [1,2,3], Sara Asghari Dilmani [1,2], Ahmed Choukri Abdullah [4], Mutlu Sarıkaya [5], Defne Yigci [3], Gozde Korkmaz [2,3] and Savas Tasoglu [1,2,4,6,7,8,*]**

[1]School of Biomedical Sciences and Engineering, Koç University, 34450 Istanbul, Turkey
[2]Koç University Translational Medicine Research Center (KUTTAM), Koç University, 34450 Istanbul, Turkey
[3]School of Medicine, Koç University, 34450 Istanbul, Turkey
[4]Department of Mechanical Engineering, Koç University, 34450 Istanbul, Turkey
[5]Department of Biochemistry Faculty of Pharmacy, Ankara University, 06560 Ankara, Turkey
[6]Koç University & Is Bank Artificial Intelligence Center (KUIS AI), Koç University, 34450 Istanbul, Turkey
[7]Koç University Arçelik Research Center for Creative Industries (KUAR), Koç University, 34450 Istanbul, Turkey
[8]Boğaziçi Institute of Biomedical Engineering, Boğaziçi University, 34684 Istanbul, Turkey
* Correspondence: stasoglu@ku.edu.tr

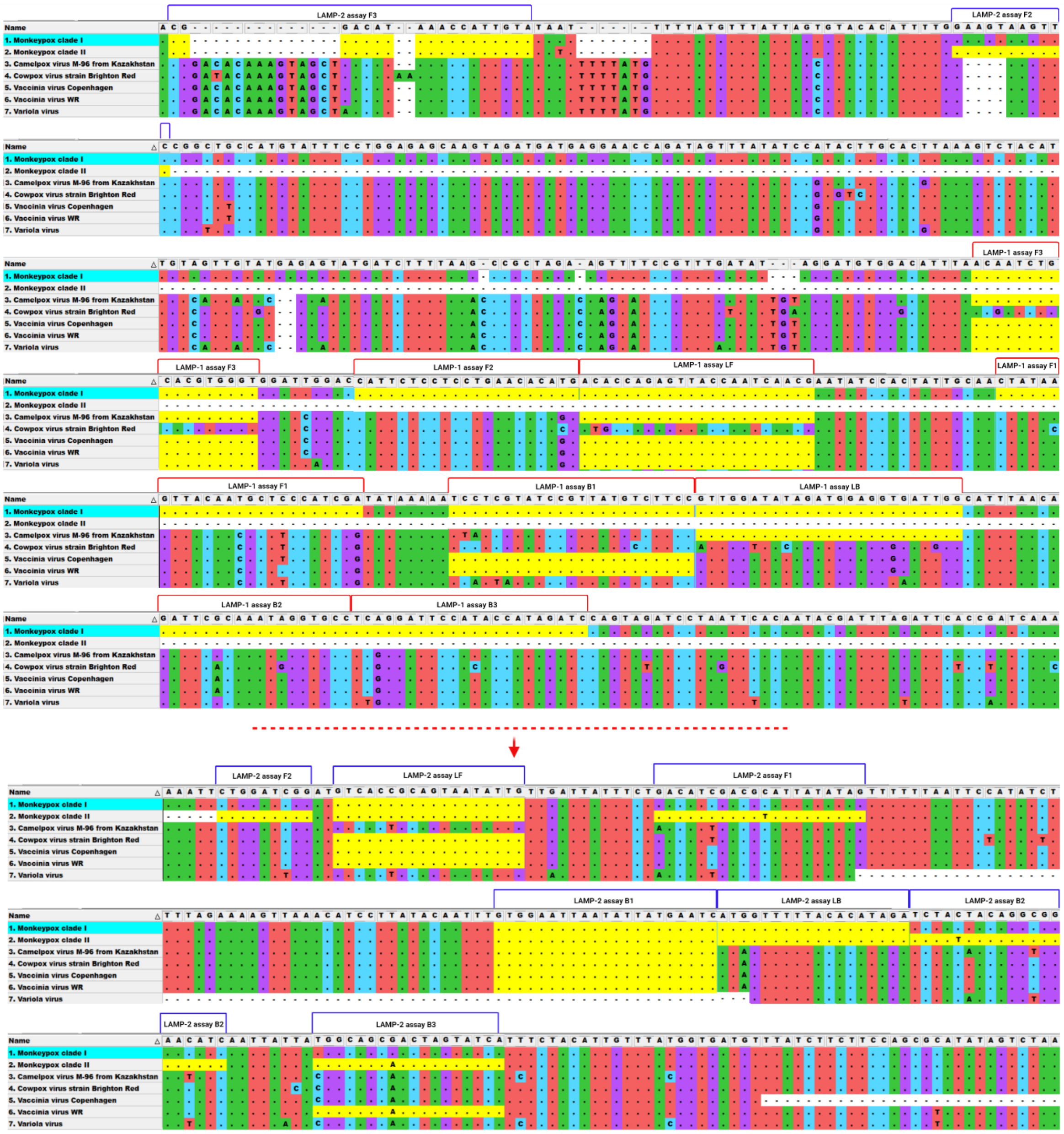


**Supp Figure S1** Genetic map matching with MPXV-Clades and *Poxviridaes* family members and primer locations of LAMP assays on the map. The deletion found in Clade-II is not found in other Poxviridae family members. The LAMP-2 assay (R- Duplex DARQ-2 probe) can distinguish Clade-II from both Clade-I and *Poxviridae* family members (white-marked regions indicate deletions). All primers for the LAMP-1 assay were selected from the region present in the Clade-I genome sequence but deleted in Clade-II. LAMP-1 assay primer regions show nucleotide differences in different regions from *Poxviridae* family members.

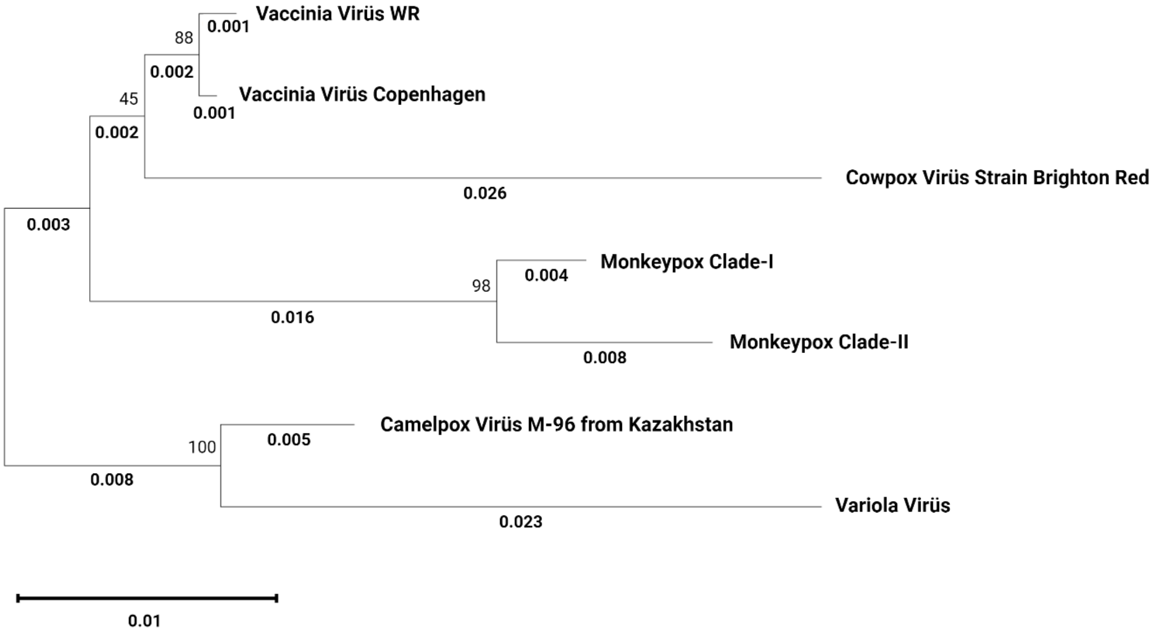


**Supp Figure S2** Phylogenetic tree represents the evolutionary relationships of Monkeypox Clade-I with other *Orthopoxvirus* species. Vaccinia viruses and Cowpox virus are relatively close relatives to MPXV Clade-I, split by a genetic distance of 0.026 and 0.004, respectively. The genetic distance between MPXV Clade-II and Clade-I is 0.008. MPXV Clade-I separates Camelpox and Variola viruses by a genetic distance of 0.016 and 0.023, respectively, indicating more distantly related relationships.

**Supp Table S1** Orthologous gene region mapping between MPXV-Clades and *Orthopoxvirus* family members and nucleotide locations of the region used in the phylogenic analysis

| Virus Name | Genbank ID | NCBI ID | Position |
|---|---|---|---|
| Monkeypox_zaire 96-I-16 (Clade-I) | AF380138 | NC_003310.1 | 18701-21151 |
| MPXV-M5312_HM12_Rivers (Clade-II) | MT903340 | NC_063383.1 | 18651-19150 |
| Vaccinia virus WR (Western Reserve) | AY243312. | NC_006998.1 | 18433-20913 |
| Vaccinia virus Copenhagen | M35027.1 | | 21516-23966 |
| Camelpox virus M-96 from Kazakhstan | AF438165.1 | NC_003391.1 | 21230-23717 |
| Cowpox virus Brighton Red | AF482758.2 | NC_003663.2 | 34878-37351 |
| Variola virus India-1967, ssp. major | X69198.1 | NC_001611.1 | 11358-13755 |
